# Evolution of Self-Assembled Au NPs by Controlling Annealing Temperature and Dwelling Time on Sapphire (0001)

**DOI:** 10.1186/s11671-015-1200-0

**Published:** 2015-12-24

**Authors:** Jihoon Lee, Puran Pandey, Mao Sui, Ming-Yu Li, Quanzhen Zhang, Sundar Kunwar

**Affiliations:** College of Electronics and Information, Kwangwoon University, Nowon-gu, Seoul 139-701 South Korea; Institute of Nanoscale Science and Engineering, University of Arkansas, Fayetteville, AR 72701 USA

**Keywords:** Au nanoparticle, Au piles, Nano-mounds, Annealing temperature, Dwelling time

## Abstract

Au nanoparticles (NPs) have been utilized in a wide range of device applications as well as catalysts for the fabrication of nanopores and nanowires, in which the performance of the associated devices and morphology of nanopores and nanowires are strongly dependent on the size, density, and configuration of the Au NPs. In this paper, the evolution of the self-assembled Au nanostructures and NPs on sapphire (0001) is systematically investigated with the variation of annealing temperature (AT) and dwelling time (DT). At the low-temperature range between 300 and 600 °C, three distinct regimes of the Au nanostructure configuration are observed, i.e., the vermiform-like Au piles, irregular Au nano-mounds, and Au islands. Subsequently, being provided with relatively high thermal energy between 700 and 900 °C, the round dome-shaped Au NPs are fabricated based on the Volmer-Weber growth model. With the increased AT, the size of the Au NPs is gradually increased due to a more favorable surface diffusion while the density is gradually decreased as a compensation. On the other hand, with the increased DT, the size and density of Au NPs decrease due to the evaporation of Au at relatively high annealing temperature at 950 °C.

**Electronic supplementary material:**

The online version of this article (doi:10.1186/s11671-015-1200-0) contains supplementary material, which is available to authorized users.

## Background

**Fig. 1:**
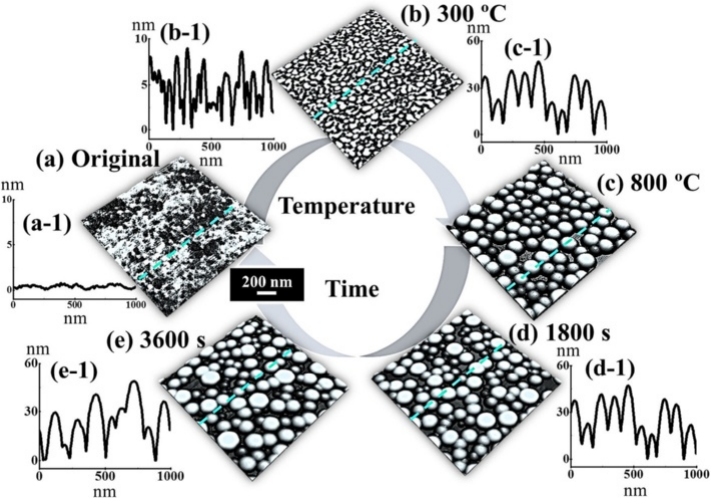
Illustration of the evolution of self-assembled Au nanostructures and nanoparticles (NPs) by the control of annealing temperature (AT) and dwelling time (DT) on sapphire (0001). **a** Three dimensional (3-D) atomic force microscopy (AFM) side view of the pre-annealed sample surface. **b**–**c** 3-D AFM side views of Au nanostructures fabricated with 3-nm deposition annealed at 300 and 800 °C with the DT of 450 s. **d**–**e** 3-D AFM side views of Au NPs with 3-nm of deposition amount (DA) annealed at 950 °C for the DT of 1800 and 3600 s. (*a-1*)–(*e-1*) Cross-sectional line profiles of the corresponding samples indicated with the *dotted lines*. **a**–**e** 1 × 1 μm^2^

Metallic nanoparticles reflect highly enhanced properties accordingly with the variation of their shape, size, and density, and thereby, various devices have been fabricated based on them [1–7]. For instance, a star-shaped Au nanoparticle (NP) deposited on quantum dot solar cells demonstrated an enhanced performance of solar cells because of its broadband scattering and absorption cross sections [7]. In addition, the Au NPs have exhibited great potentials as a catalyst for the fabrications of nanopores [8–12] and nanowires [13–17]. With the Au NP-assisted etching, the nanopores can be acquired by desorbing the underlying substrate such as in silicon [8, 9] and silicon oxide [10]. Therefore, the size, density, and even the shape of the nanopores can strongly depend on the size, density, and shape of the Au NPs. Similarly, in the fabrication of the nanowires, the liquid-phase Au NPs act as the catalyst to absorb the target atoms in vapor phase until reaching the super-saturation. Then, the corresponding nanowires are nucleated at the solid-liquid interface via the vapor-liquid-solid (VLS) mechanism [13–17]. Therefore, the size, density, and shape of the nanowires are directly determined by the diameter, density, and shape of the Au NPs. Meanwhile, owing to the high chemical stability [18, 19], wide bandgap, and high thermal tolerance [20], sapphire has been widely adapted in optical and optoelectronic devices such as optical lenses [18, 19] and LEDs [21]. In the previous works, various metal films had been studied on oxide surfaces [22–25]. For instance, Beszeda et al. demonstrated the kinetics of Ostwald ripening occurring between 350 and 500 °C and the kinetics of evaporation observed above 670 °C during the ultra-high vacuum annealing, investigated by the auger electron spectroscopy [22]. Other experiments on the metal NPs on oxide surfaces demonstrate their various properties [23], morphology [24], and evolution of Au nanostructures as a function of film thickness [25], but the systematic control of the size, shape, and density of metal NPs with the variation of annealing temperature and dwelling time was not reported. This is distinctive from the previous reports and is beneficial to investigate the systematic fabrication of the Au NPs on sapphire, which is still deficient. In this paper, we investigate the evolution of the self-assembled Au nanostructures on sapphire (0001) by the control of annealing temperature (AT) and the dwelling time (DT). The shape and size evolution of the Au NPs on sapphire was observed between 300 and 900 °C due to the favorable Au adatom diffusion, and the evaporation occurred only at 950 °C, and the rate of evaporation increased with the increased dwelling time, characterized by the atomic force microscopy (AFM) and energy-dispersive X-ray spectroscopy (EDS). In recent study of Au NPs on GaAs, with the variation of deposition amount (2 to 20 nm), annealing temperature (250 to 550 °C), and dwelling time (450 to 3600 s), the size of Au NPs increases whereas the shape remains dome [26]. Depending upon the substrate materials and growth condition, the evolution of Au NPs shows variant behaviors so we observed the Au piles, nano-mounds, islands, and the size evolution in this paper. Figure 1 shows the illustration of Au nanostructure evolution with the control of the AT and DT. In general, the Au nanostructures including the Au piles, nano-mounds, islands, and the self-assembled round dome-shaped Au NPs were successfully fabricated on sapphire by varying the AT with the fixed deposition amount (DA) and DT, as shown in Fig. 1b, c. The evolution of the Au nanostructures was attributed to the enhanced diffusion length which is due to the increased surface diffusion of the Au adatoms at elevated AT. In addition, when the AT was increased beyond 700 °C, with a more favorable surface diffusion, the size of the Au NPs was gradually increased while the density of the Au NPs was decreased as the compensation. Meanwhile, the increased DT showed a mild effect on the evolution of the Au NPs, as shown in Fig. 1d, e.

## Methods

### Substrate Preparation

In this experiment, the size, density, and configuration of the self-assembled Au nanostructures were investigated with the variation of annealing temperature and dwelling time. At the beginning of the experiment, a 430-μm-thick sapphire (0001) substrate with off-axis ±0.1^o^ from the iNexus Inc. (South Korea) was cleaved. After the RCA cleaning, the substrate was indium-bonded to an Inconel holder and introduced in a pulsed laser deposition (PLD) chamber for degassing at 350 °C for 1800 s under 1 × 10^−4^ Torr to remove the contaminants. Additional file 1: Figure S1 shows the Raman spectra of bare sapphire in the range between 154 and 1388 cm^−1^ excited by a continuous wave (CW) laser of 532 ± 1 nm wavelength at room temperature. Additional file 2: Figure S2 shows the AFM image and line profile of the bare sapphire (0001) and its crystal structure.

### Sample Growth

In this work, the effects of AT between 300 and 900 °C as well as the DT from 150 to 3600 s on the evolution of the Au NPs were systematically investigated by annealing the samples in a PLD system. After degassing, the samples were deposited on a 3-nm Au film, at a growth rate of 0.05 nm/s with the ionization current of 3 mA under the vacuum of 1 × 10^−1^ Torr in an ion-coater sputter. Subsequently, in order to study the AT effect with the fixed DA and DT, the sample temperature was systematically ramped up to each target temperature (300, 400, 500, 600, 700, 800, 850, 900, 950) in a PLD chamber at a ramping rate of 1.83 °C/s under 1 × 10^−4^ Torr with the computer-operated recipes, and then the samples were dwelt at the target temperature of 450 s to ensure the heat conduction. After that, the sample temperature was immediately quenched down to the ambient temperature to avoid further Ostwald ripening. In order to investigate the effect of the DT on the resulting Au NPs with the identical AT, diverse DTs of 150, 1800, and 3600 s were dwelt when the samples reached the target AT of 950 °C with the DA of 3 nm.

### Characterization of Au Nanostructures and NPs

After the successful growth of each sample, the surface morphology was investigated utilizing an AFM. The AFM with a non-contact mode was used to characterize the sample surface morphologies. The tips used in the AFM has a height of 17 μm with a radius curvature of ~10 nm, a force constant of 40 N/m, and a resonant frequency of ~270 kHz. In order to minimize the tip effect on the measurement of the resulting Au NPs, the tips utilized were from the same batch. The acquired data were analyzed by XEI software (Park Systems) to investigate the size and density of observed Au NPs in terms of the AFM images, 2-D Fourier filter transform (FFT) power spectra, and cross-sectional line profiles. Also, an EDS system (Thermo Fisher Noran System 7) was employed to perform the elemental analysis under the vacuum. Additionally, the Raman spectrum of the bare sapphire was acquired by a charge-coupled device (CCD) detector excited by a CW laser with the wavelength of 532 ± 1 nm.

## Results and Discussion

**Fig. 2:**
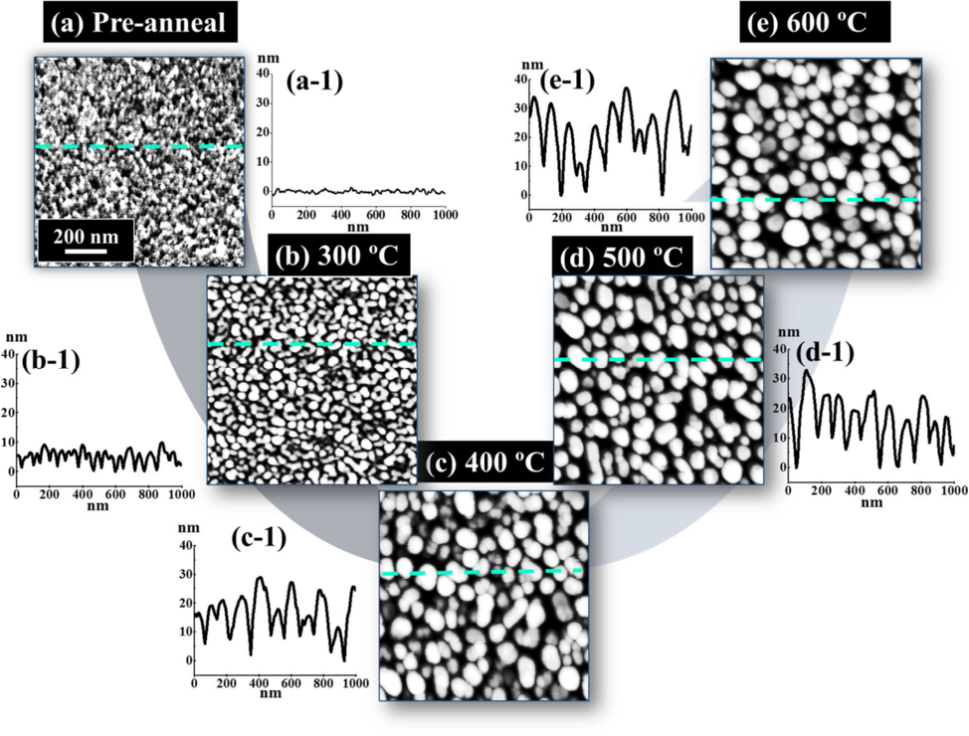
Effect of the annealing temperature (AT) on the Au nanostructure evolution with 3 nm of DA and 450 s of DT. **a**–**e** AFM top views (1 × 1 μm^2^) of the pre-annealed sample surface and the Au nanostructures annealed at 300, 400, 500, and 600 °C, respectively. (*a-1*)–(*e-1*) Cross-sectional line profiles of the corresponding samples

**Fig. 3:**
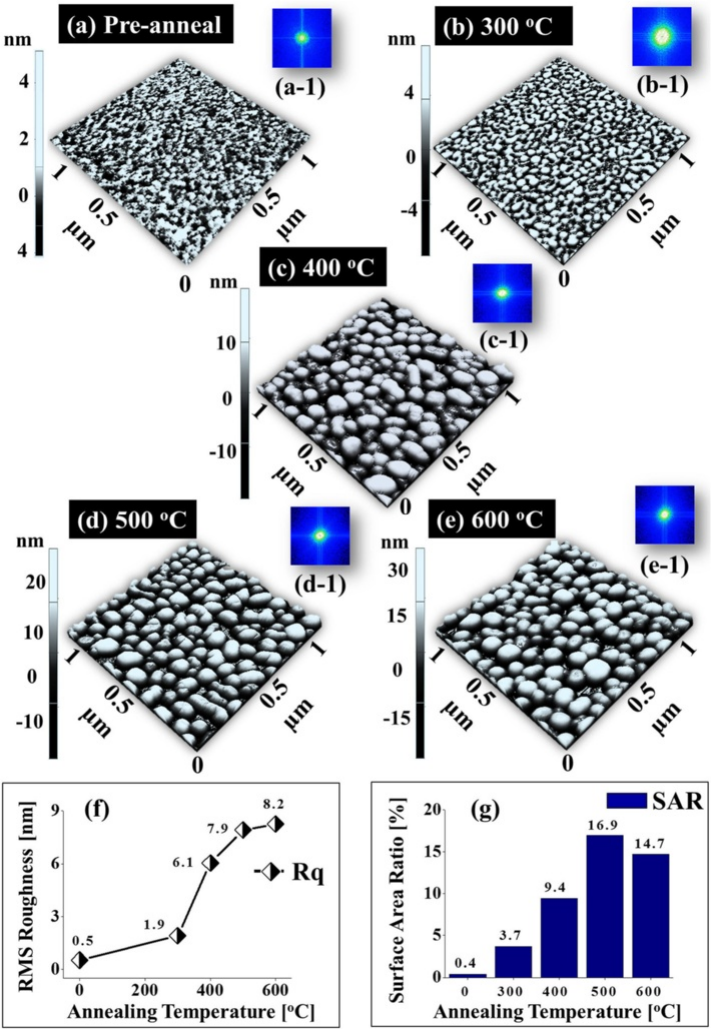
AFM side views, FFT power spectra, and plots of RMS roughness and surface area ratio of corresponding Fig. 2. The color scale panels near the AFM images indicate the height variation

Figure 2 shows the evolution of the self-assembled Au nanostructures on sapphire (0001) by varying the AT between 300 and 600 °C with the DA of 3 nm and the DT of 450 s. In general, with the increased AT, the configuration of the nanostructure has changed as well as the size of Au nanostructures increased whereas the density decreased. At first, as shown in Fig. 2a, the pre-annealed surface shows the uniform distribution of Au films all over the substrate surface but when the annealing was performed, the Au adatoms aggregate and the vermiform Au piles were formed as presented in Fig. 2b. Further increase in annealing temperature resulted in the formation of Au nano-mounds and the size was gradually increased, as shown in Fig. 2c, d, and ultimately, the increased size and quite uniform distribution of Au islands were observed as shown in Fig. 2e. The variation in shape, size, and density of Au nanostructures and NPs can be illustrated based on the dewetting mechanism and diffusion theory. In the dewetting mechanism, the continuous thin Au films break during the annealing below the melting point, and thus, the Au adatoms aggregate to form the Au NPs minimizing the overall surface energy. The rate of the dewetting is enhanced with the increased annealing temperature due to the temperature dependence surface diffusion of the Au adatoms. The surface diffusion coefficient (*D*_S_) is positively correlated with the AT, *D*_s_ ∝ exp(−*E*_n_/*KT*_a_), where *K* is the Boltzmann constant, *E*_n_ is the diffusion barrier, and *T*_a_ is the annealing temperature [27]. Also, the relationship between the diffusion length *L*_D_ and the surface diffusion coefficient *D*_S_ can be acquired from the equation *L*_D_ = √ (*D*_S_*t*), where *t* is the residence time. Thus, the diffusion length *L*_D_ is directly determined by the surface diffusion coefficient *D*_S_. Therefore, the diffusion length of the Au adatoms is strongly dependent upon the annealing temperature. As a result, the Au adatoms diffused more drastically with the increased annealing temperature. Initially, with 3-nm Au deposition and pre-annealing, the surface morphology was uniform as clearly shown by the cross-sectional line profile in Fig. 2 (a-1). After 300 °C of annealing, the deposited Au film diffused and aggregated as a vermiform Au pile due to a short diffusion length based on the diffusion-limited aggregation (DLA) [28]. The irregular-sized Au islands were clearly observed as shown by AFM top-view and cross-sectional line profile in Fig. 2b (b-1). The similar configuration of Au piles was also observed in previous research such as Au on glass, Si with thermally grown oxide, and pentacene organic thin films on SiO_2_ [29–31]. The increase in annealing temperature to 400 °C resulted in the slightly increased diffusion length such that the Au adatoms can aggregate more compactly leading to the formation of Au nano-mounds. The size of Au nano-mounds increased and density was decreased as clearly shown in Fig. 2c (c-1). Similarly, at 500 °C, due to the favorable diffusion length, the Au nano-mound size slightly increased whereas the density decreased as evidenced by the cross-sectional line profile in Fig. 2 (d-1). At 600 °C of annealing, the sizes of Au islands are quite uniformly distributed and are increased compared with the preceding samples as shown in Fig. 2 (e-1). Figure 3 shows the AFM side views, FFT power spectra, and plots of RMS roughness and surface area ratio of the corresponding samples. The size, density, and configuration of Au nanostructures also can be described on the basis of FFT power spectra. As shown by the FFT power spectra in Fig. 3 (a-1), the - nm Au deposited shows the small round pattern with the uniform distribution of Au. With the increased AT, the irregular height distribution of Au nanostructures lead to the big and bright pattern of FFT power spectra in Fig. 3 (c-1). Due to the slightly improved height distribution uniformity, the FFT power spectra become slightly smaller and dim as shown in Fig. 3 (d-1) and, in the same manner, the pattern is small and dim in Fig. 3 (e-1). Similarly, the increase in the Rq and SAR also illustrated the gradually increased size of Au NPs as clearly shown in Fig. 3f, g. The Rq was increased from 0.5 to 8.2 nm with the increased annealing temperature from the pre-annealing to 600 °C. This is due to the variant morphology of the Au nanostructures at different annealing temperature. Similar to our results, the Rq of the GaN layer grown on SiC substrates at various temperatures between 975 and 1130 °C showed the increased Rq with the increased temperature [32].

**Fig. 4:**
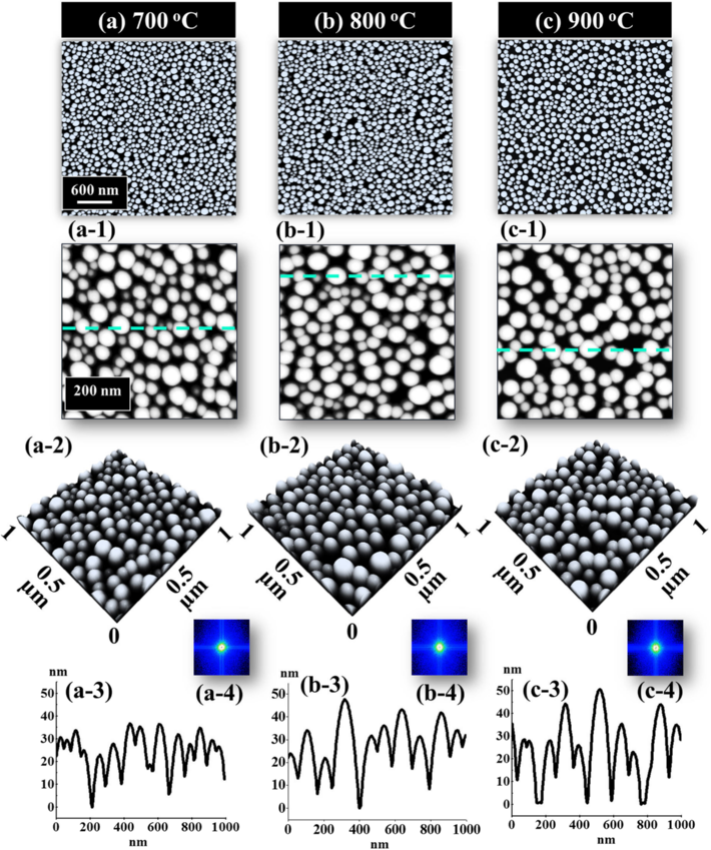
Evolution of the self-assembled Au NPs with the variation of annealing temperature between 700 and 900 °C. **a**–**c** AFM top views of 3 × 3 μm^2^. (*a-1*)–(*c-1*) AFM top views of 1 × 1 μm^2^. (*a-2*)–(*c-2*) Corresponding side views. (*a-3*)–(*c-3*) Cross-sectional line profiles. (*a-4*)–(*c-4*) FFT power spectra

**Fig. 5:**
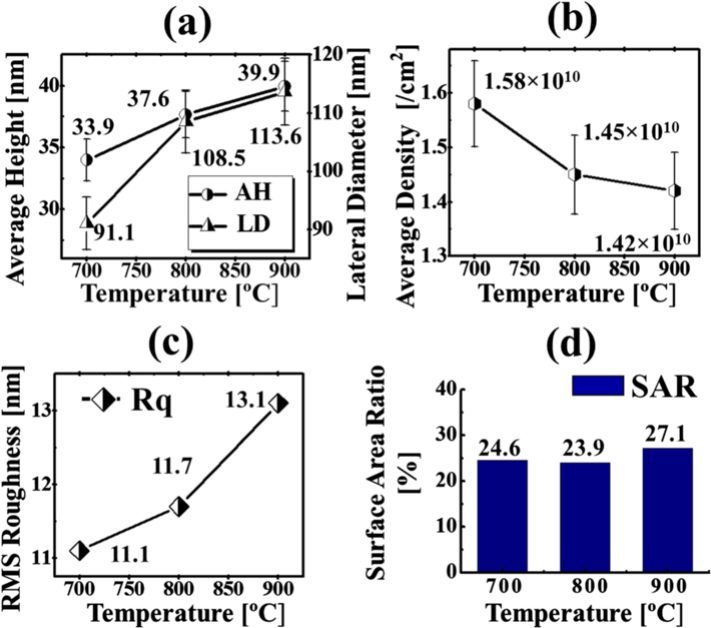
Summary plots of the (**a**) average height (AH) and lateral diameter (LD), (**b**) average density (AD), (**c**) root-mean-squared (RMS) roughness (Rq), and (**d**) surface area ratio (SAR) of the Au NPs. *Error bars* ± 5 % in (**a**) and (**b**)

Figures 4 and 5 show the evolution of the self-assembled Au NPs with the variation of annealing temperature from 700 to 900 °C. The AFM top-view, side-view, cross-sectional line profiles and FFT power spectra are shown in Fig. 4 while the plots of average height (AH), average lateral diameter (LD), average density (AD), RMS roughness, and surface area ratio of the corresponding samples are presented in Fig. 5. The side-views of 3 × 3 µm2 AFM images are provided in Additional file 3: Figure S3. In general, on the basis of increased annealing temperature, the size of self-assembled NPs increased and the uniformity of height distribution of NPs slightly improved whereas the density was decreased. When the AT was further increased beyond 600 °C, with sufficient thermal energy provided, the surface diffusion and diffusion length is also increased, and as a result, the isolated Au islands evolved into the self-assembled round dome-shaped Au NPs based on the Volmer-Weber growth model [33–35], as shown in Fig. 4. As the thermal energy is sufficient, the diffusion length is also enough. Also, the bonding energy of Au adatoms are stronger than that of Au adatoms and sapphire surface atoms, i.e., the Au adatom-cohesive force is stronger than that of sapphire surface-adhesive force, and as a result, the Au adatoms aggregated in the form of 3-D self-assembled Au NPs. Therefore, with the increased annealing temperature, the size evolution of the Au NPs can be attributed to the large self-assembled Au NPs absorbing the nearby smaller ones in order to lower the surface energy which is also evidenced by the summary plots in Fig. 5a–d. When the sample was annealed at 700 °C, the self-assembled Au NPs appeared with the average height (AH) of 33.9 nm, the average lateral diameter (LD) of 91.1 nm, and the average density (AD) of 1.58 × 10^10^ cm^−2^. When the AT was increased to 800 °C, owing to the increased diffusion length of the Au adatoms, relatively larger Au NPs absorbed the adjacent smaller ones. As a result, the AH of the self-assembled Au NPs increased by 1.11 times to 37.6 nm, and the LD increased by 1.19 times to 108.5 nm, while the AD decreased by 1.08 times to 1.45 × 10^10^ cm−^2^ as the compensation. Also, it is clearly observed from the AFM images in Fig. 4a, b that the AD of Au NPs was decreased with the increased AT. Likewise, when the sample was annealed at 900 °C, the AH and LD of the Au NPs slightly increased by 1.06 and 1.05 times to 39.9 and 113.6 nm, respectively. As a compensation, the AD was decreased by 1.02 times to 1.42 × 10^10^ cm^−2^. Similar behavior was observed with the samples at 850 and 950 oC as shown in Additional file 4: Figure S4 and Additional file 5: Figure S5. On the other hand, during the evolution of the Au NPs from 700 to 900 °C, the uniformity of the self-assembled Au NPs was gradually improved, as shown by cross-sectional line profiles in Fig. 4 (a-3) (c-3). As evidenced by the size evolution of Au NPs with increasing AT, the bright spots in the FFT power spectra became small and dim, as shown in Fig. 4 (a-4) (c-4). In addition, the RMS roughness of the self-assembled Au NPs increased from 11.1 to 13.1 nm and surface area ratio from 24.6 to 27.1 %, when the AT was increased from 700 to 900 °C, which was induced by the formation of the relatively larger Au NPs. The evolution of size and reduction of NPs density was also observed with metallic NPs on various substrate surfaces. Hence, a similar evolution trend of the Au NPs was also observed on GaAs, Si [36–38], and Pt NPs on Si [39].

**Fig. 6:**
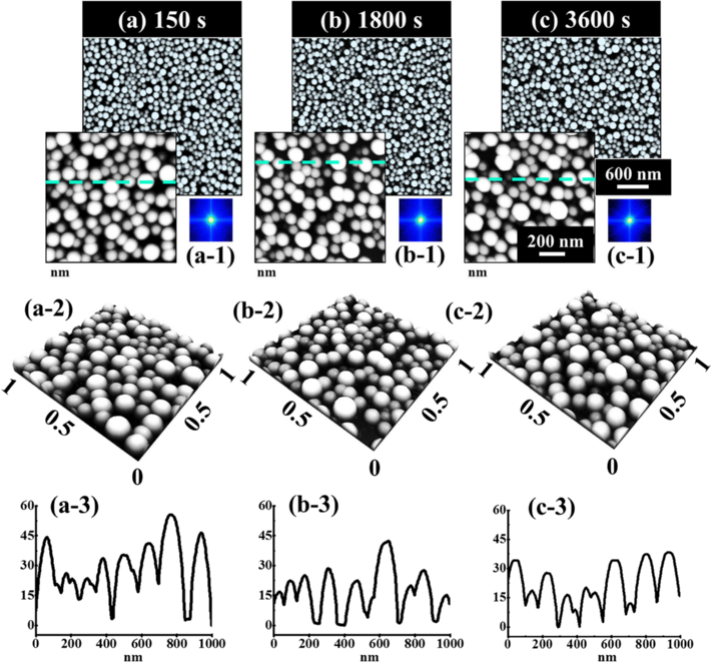
Effects of the dwelling time on the fabrication of the self-assembled Au NPs annealed at 950 °C with the DA of 3 nm. **a**–**c** AFM top views of 3 × 3 μm^2^ in which the insets are of 1 × 1 μm^2^. (*a-1*)–(*c-1*) FFT power spectra. (*a-2*)–(*c-2*) AFM side views. (*a-3*)–(*c-3*) Cross-sectional line profiles of the corresponding samples

**Fig. 7:**
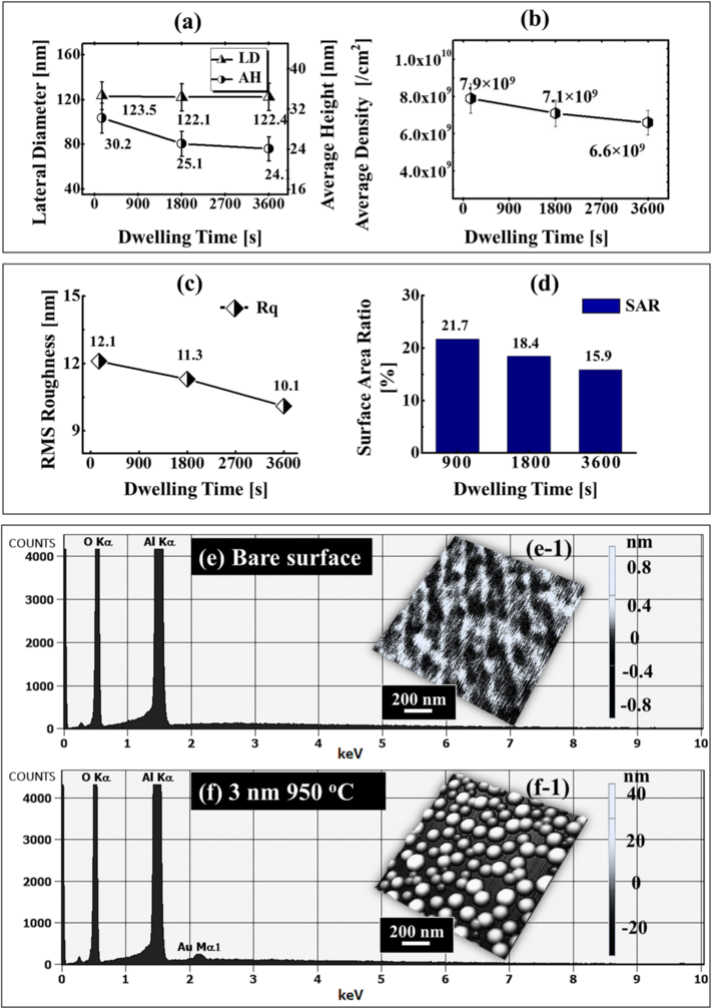
**a**–**d** Plot of AH, LD, AD, Rq, and SAR of the Au NPs with diverse DTs. **e**–**f** Energy-dispersive X-ray spectroscopy (EDS) spectra of the sapphire bare surface and the sample with DA of 3 nm annealed at 950 °C. (*e-1*)–(*f-1*) 3-D AFM side views of 1 × 1 μm^2^. *Error bars* ± 5 % in (**a**) and (**b**)

Figure 6 shows the effect of dwelling time for the fabrication of self-assembled Au NPs on sapphire. In order to investigate the effect of DT on the self-assembled Au NPs evolution, the samples were annealed at 950 °C for diverse DTs of 150, 1800, and 3600 s with the DA of 3 nm, as shown by AFM top-view, cross-sectional line profiles and FFT power spectra in Fig. 6 and plots and EDS spectra in Fig. 7. Corresponding 3 × 3 µm2 AFM side views are shown in Additional file 6: Figure S6 It is observed that the variation of dwelling time slightly affects the morphology of self-assembled Au NPs compared with the annealing temperature. In general, with the increased dwelling time, the size of metal NPs increase and density decrease but in our case, due to the high temperature of annealing, the evaporation of Au led to the decreased size and density simultaneously. The size and density evolution of the Au NPs can be described based on the Ostwald ripening, namely, the Au adatoms being detached from the smaller Au droplets are attached to the larger ones [40–42]. In the process of the Ostwald ripening, the Au adatoms were detached from the smaller Au NPs and formed the monomers on the substrate. Subsequently, the monomers were attached to the relatively large Au NPs [40]. In addition, the driving force for the Au adatoms leaving the substrate and being attached to the larger Au NPs was the difference of the chemical potential between the existing Au NPs with the radius *R* and the NPs with the critical radius R* [40, 41]. Herein, the Au NPs with the critical radius R* means that the Au NPs were at equilibrium mode that neither absorbed nor released the Au adatoms. For example, on the one hand, when the radius *R* of the existing Au NPs was bigger than the critical radius R*, the bigger Au NPs with the lower chemical potential tended to absorb the Au adatoms. As a result, the Au adatoms can be absorbed to the existing large Au NPs from the substrate surface, resulting in the further increased size of the larger Au NPs [41, 42]. Thus, the Au adatoms were attached to the substrate surface from the smaller Au NPs, which leads to shrinkage and disappearance of smaller Au NPs, and as a result, the density of NPs decreased. From the plot of average density in Fig. 7b, we can observe that the density is gradually decreased with the increased dwelling time. Initially, at 150 s, the AD was 7.9 × 10^9^ cm^−2^, and it decreased by 1.11 time to 7.1 × 10^9^ cm^−2^ at 1800 s and further decreased by 1.08 times to 6.6 × 10^9^ cm^−2^ at 3600 s of dwelling time. Along with the decreased density, the size of NPs also decreased accordingly with the increased dwelling time. The reduction of size may be caused by the evaporation of Au at high temperature of annealing such that when the dwelling time increases, the rate of evaporation can also increase accordingly with the increased dwelling time [43]. Consequently, when the sample was annealed at 950 °C for 150 s, the AH and LD of the Au NPs were 30.2 and 123.5 nm, respectively, as shown in Fig. 7a. As the DT was increased to 1800 s, the AH slightly decreased by 1.2 times to 25.1 nm and LD by 1.01 times to 122.1 nm. Similarly, when the DT was further increased to 3600 s, the AH slightly decreased by 1.04 to 25.1 nm whereas LD almost remains constant to 122.4 nm, as clearly shown by the plot in Fig. 7a. The RMS roughness and surface area ratio of the corresponding samples also evidenced the morphology of the Au NPs as shown in Fig. 7c, d. The (EDS) spectra of the sapphire bare surface and the sample with 3-nm DA annealed at 950 °C are shown in Fig. 7e, f. From the EDS spectrum in Fig. 7f, the Au Mα1 peak at 2.123 keV is clearly observed which evidenced the presence of Au whereas in Fig. 7e, there is no presence of such peak at 2.123-keV energy level.

## Conclusions

In this paper, the evolution of the self-assembled Au nanostructures and NPs were systematically investigated by the control of AT and DT. In general, below the 700 °C of AT, the evolution of the Au nanostructures was divided into three distinct regimes based on their configuration: the vermiform Au piles; irregular Au nano-mounds; and Au islands, and the evolution can be attributed to the enhanced surface diffusion due to the increased AT. When the AT was elevated to 700 °C, being provided with the sufficient thermal energy, the isolated Au islands evolved into the self-assembled Au NPs based on Volmer-Weber growth model due to the more favorable surface diffusion. In addition, with the increased AT between 700 and 900 °C, the size of the Au NPs was gradually increased accompanied with the decreased AD. On the other hand, with the increased DT from 150 to 3600 s, the Ostwald ripening showed a mild effect on the Au NPs evolution. The size and density of Au NPs were decreased with increased dwelling time which contradicts with the Ostwald ripening, may be due to the Au evaporation at relatively high annealing temperature (950 °C).

## Additional files

Additional file 1: Figure S1. Full range (between 154 and 1388 cm^−1^) Raman spectra of the bare sapphire (0001) surface excited by a laser of 532 ± 1 nm wavelength at the room temperature. The signal was detected by a TE cooled charge-coupled device (CCD). The two transverse optic (TO) peaks were at 375.9 and 427.7 cm^−1^, respectively, and four longitudinal optical mode (LO) peaks were shown at 413.5, 440.4, 573.4, and 747.6 cm^−1^.^1,2^ The full width at half maximum (FWHM) of the LO was 7.14 cm^−1^. (JPG 95 kb) Additional file 2: Figure S2. (**a**) AFM image of bare sapphire (0001) surface of 3 × 3 μm^2^. (a-1) Cross-sectional line profile of the sample surface. (a-2) Corresponding AFM side view. (a-3) 2-D FFT power spectra. (a-4) Crystal structure of sapphire (0001). The blue and red ball represent O and Al atoms, respectively. (JPG 255 kb) Additional file 3: Figure S3. Fabrication of self-assembled Au NPs on sapphire (0001) with the variation of annealing temperature between 700 and 900 °C. (**a**)–(**c**) 3-D AFM side view of 3 × 3 μm^2^. Scale bars indicate the height distribution of the Au NPs. (JPG 371 kb) Additional file 4: Figure S4. Self-assembled Au NPs fabricated on sapphire (0001) with the 850 and 950 °C of annealing for 450 s and 3 nm of Au DA. (**a**)–(**b**) AFM top views of 3 × 3 μm^2^, in which the insets are of 1 × 1 μm^2^. (a-1) and (b-1) Cross-sectional line profiles. (a-2)–(b-2) FFT power spectra. (JPG 269 kb) Additional file 5: Figure S5. 3-D AFM side views of the self-assembled Au NPs at 850 and 950 °C of annealing for 450 s and 3 nm of Au DA. (**a**)–(**b**) Enlarged AFM images (1 × 1 μm^2^), acquired from the green boxes in the large 3-D side views of the AFM images of 3 × 3 μm^2^ in (a-1)–(b-1). (JPG 351 kb) Additional file 6: Figure S6. Fabrication of self-assembled Au NPs by controlling the DT between 150 and 3600 s. (**a**)–(**c**) 3-D AFM side views of 3 × 3 μm^2^. (JPG 350 kb)
