# Supplementary figures and images for: Evolution of Self-Assembled Au NPs by Controlling Annealing Temperature and Dwelling Time on Sapphire (0001)
Source: Nanoscale Res Lett. 2015 Dec 24;10:494. doi: 10.1186/s11671-015-1200-0 (PMC4690826; doi:10.1186/s11671-015-1200-0)

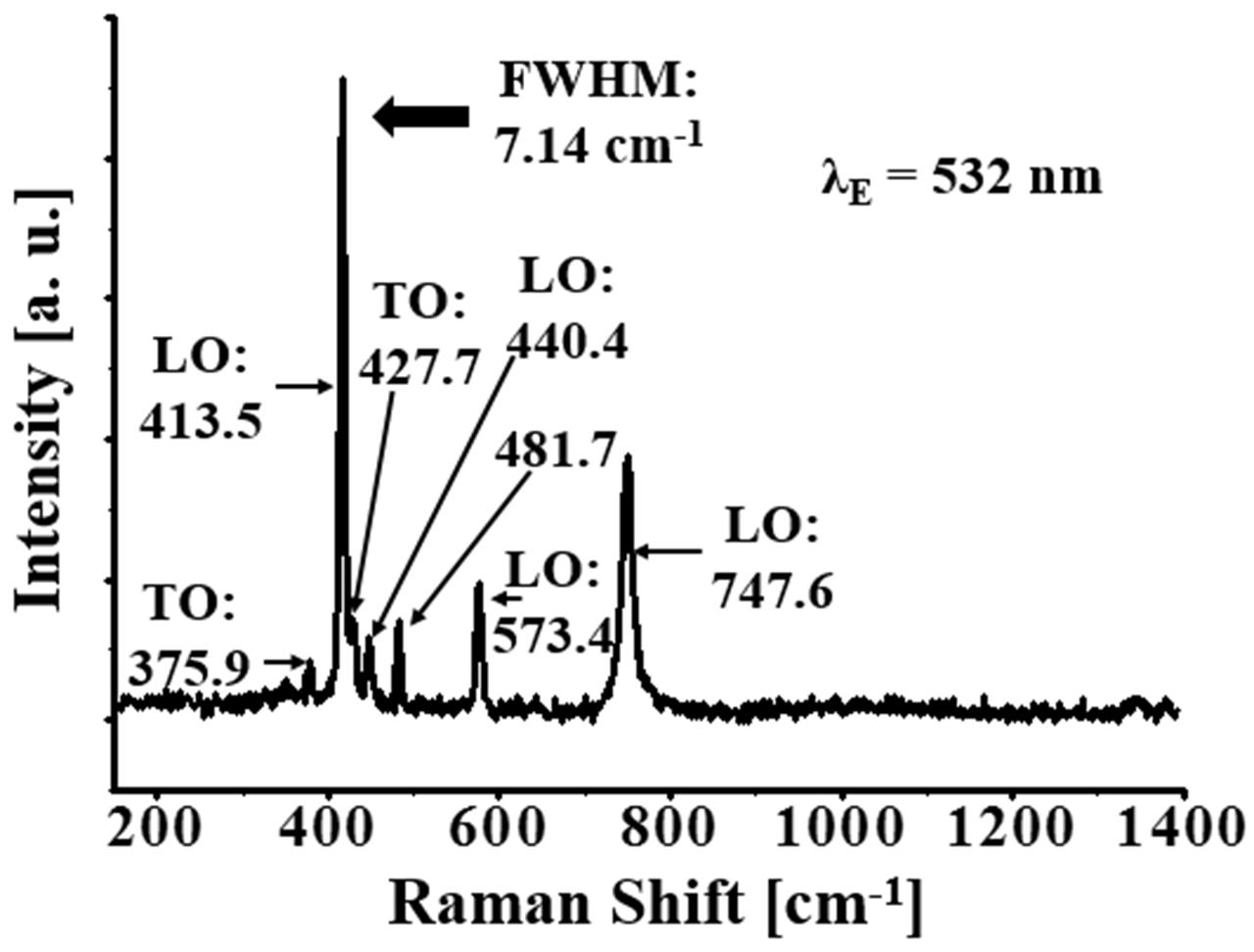

Supplement: Additional file 1: Figure S1. — Full range (between 154 and 1388 cm−1) Raman spectra of the bare sapphire (0001) surface excited by a laser of 532 ± 1 nm wavelength at the room temperature. The signal was detected by a TE cooled charge-coupled device (CCD). The two transverse optic (TO) peaks were at 375.9 and 427.7 cm−1, respectively, and four longitudinal optical mode (LO) peaks were shown at 413.5, 440.4, 573.4, and 747.6 cm−1.1,2 The full width at half maximum (FWHM) of the LO was 7.14 cm−1. (JPG 95 kb) [file 11671_2015_1200_MOESM1_ESM.jpg]

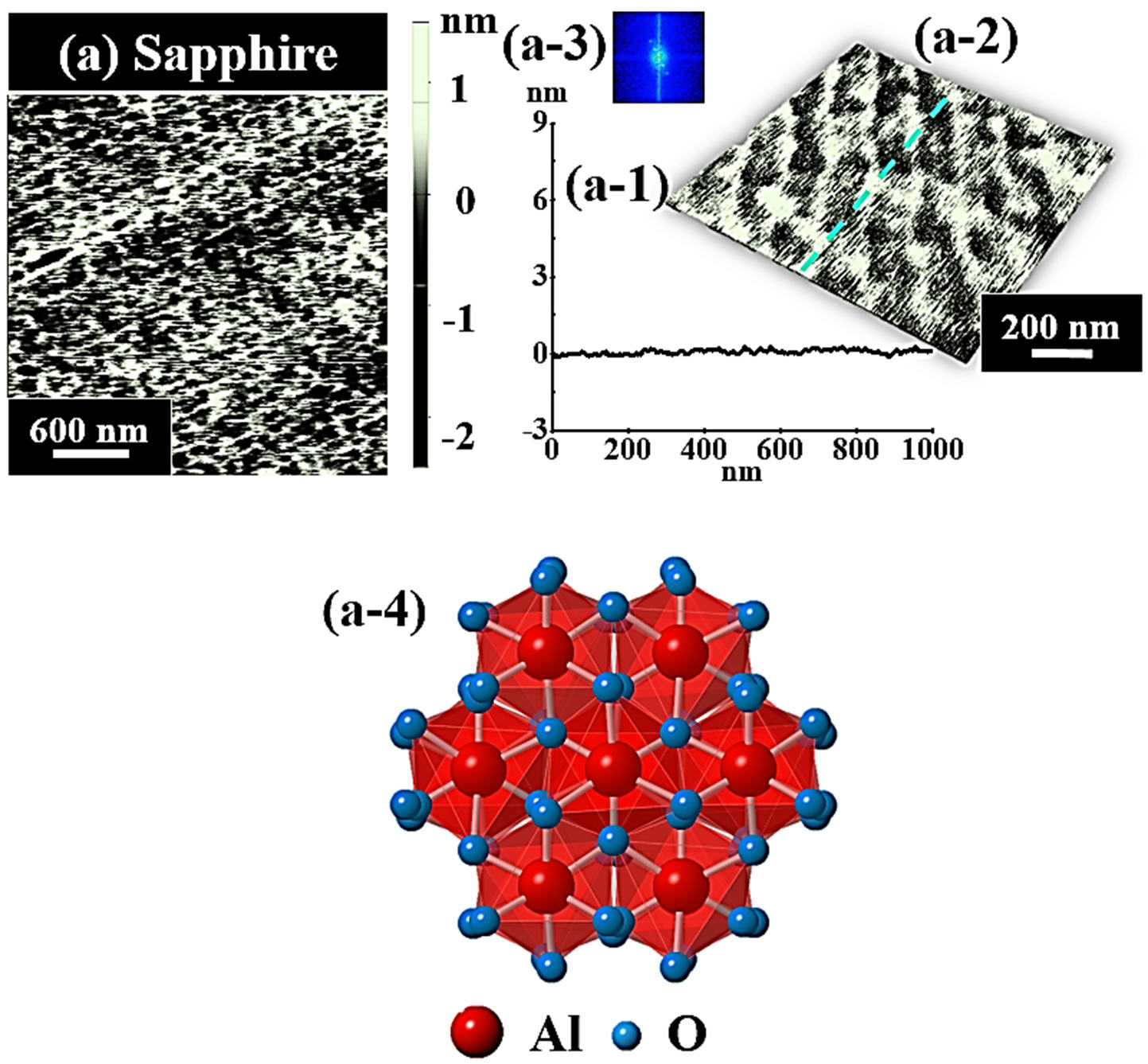

Supplement: Additional file 2: Figure S2. — (a) AFM image of bare sapphire (0001) surface of 3 × 3 μm2. (a-1) Cross-sectional line profile of the sample surface. (a-2) Corresponding AFM side view. (a-3) 2-D FFT power spectra. (a-4) Crystal structure of sapphire (0001). The blue and red ball represent O and Al atoms, respectively. (JPG 255 kb) [file 11671_2015_1200_MOESM2_ESM.jpg]

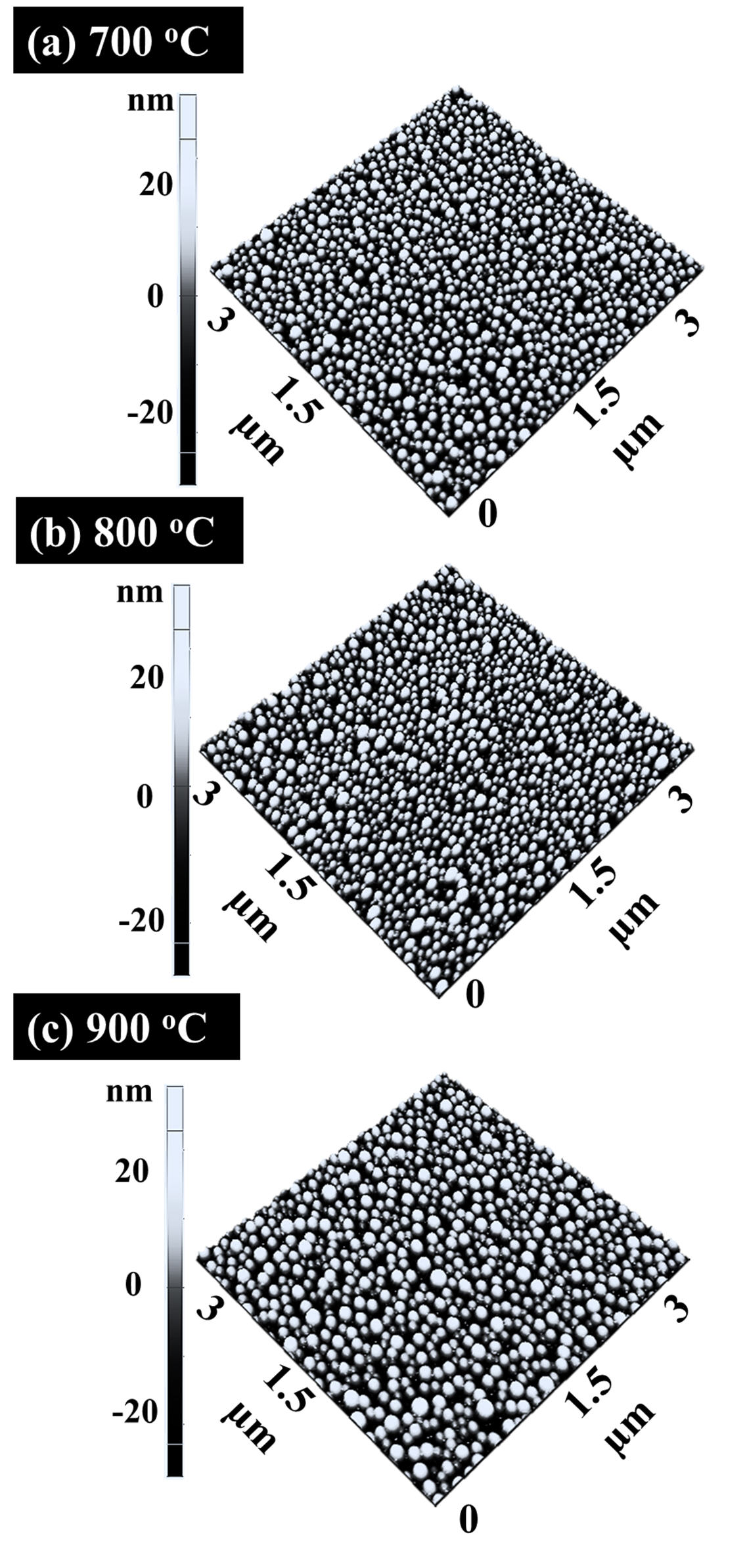

Supplement: Additional file 3: Figure S3. — Fabrication of self-assembled Au NPs on sapphire (0001) with the variation of annealing temperature between 700 and 900 °C. (a)–(c) 3-D AFM side view of 3 × 3 μm2. Scale bars indicate the height distribution of the Au NPs. (JPG 371 kb) [file 11671_2015_1200_MOESM3_ESM.jpg]

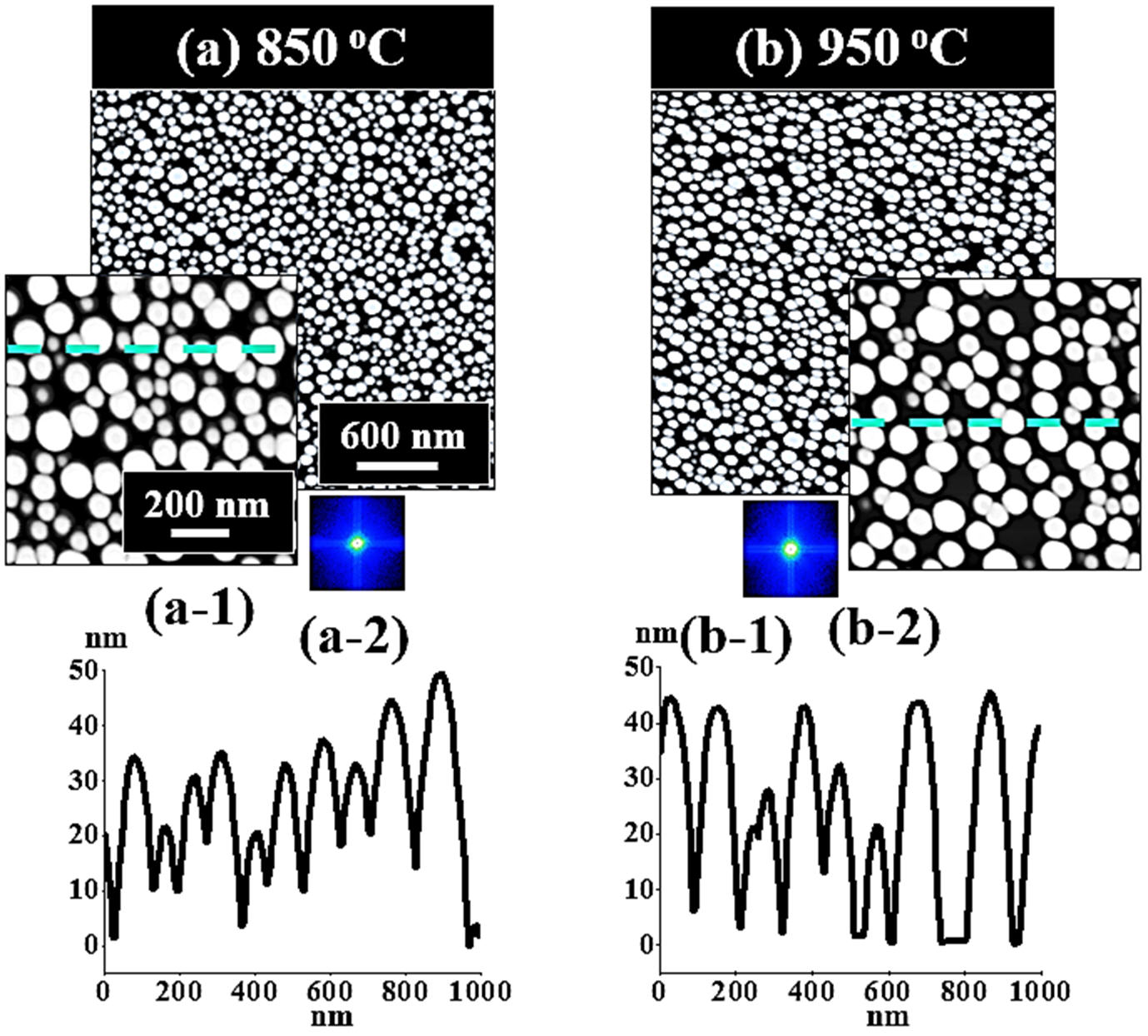

Supplement: Additional file 4: Figure S4. — Self-assembled Au NPs fabricated on sapphire (0001) with the 850 and 950 °C of annealing for 450 s and 3 nm of Au DA. (a)–(b) AFM top views of 3 × 3 μm2, in which the insets are of 1 × 1 μm2. (a-1) and (b-1) Cross-sectional line profiles. (a-2)–(b-2) FFT power spectra. (JPG 269 kb) [file 11671_2015_1200_MOESM4_ESM.jpg]

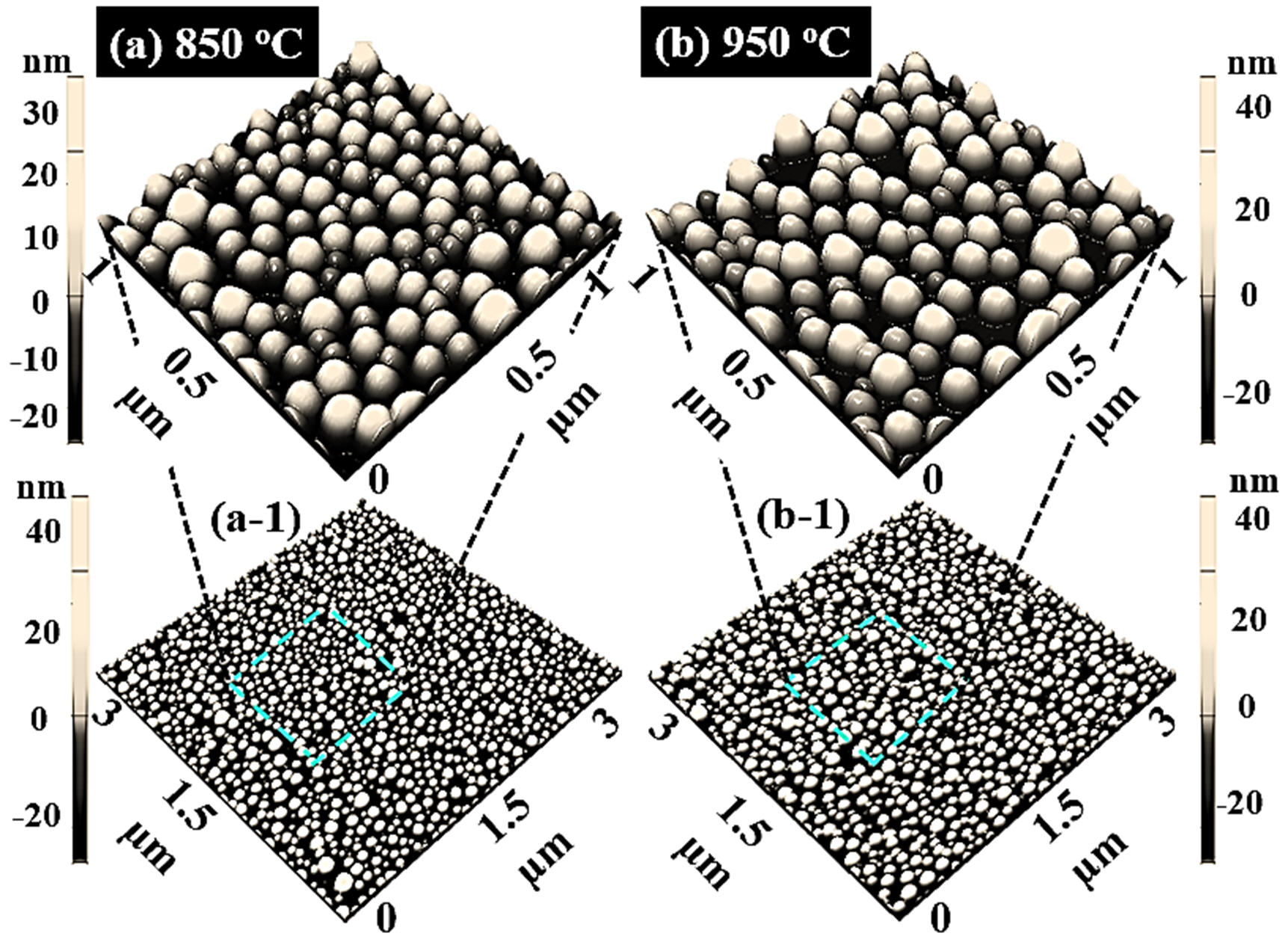

Supplement: Additional file 5: Figure S5. — 3-D AFM side views of the self-assembled Au NPs at 850 and 950 °C of annealing for 450 s and 3 nm of Au DA. (a)–(b) Enlarged AFM images (1 × 1 μm2), acquired from the green boxes in the large 3-D side views of the AFM images of 3 × 3 μm2 in (a-1)–(b-1). (JPG 351 kb) [file 11671_2015_1200_MOESM5_ESM.jpg]

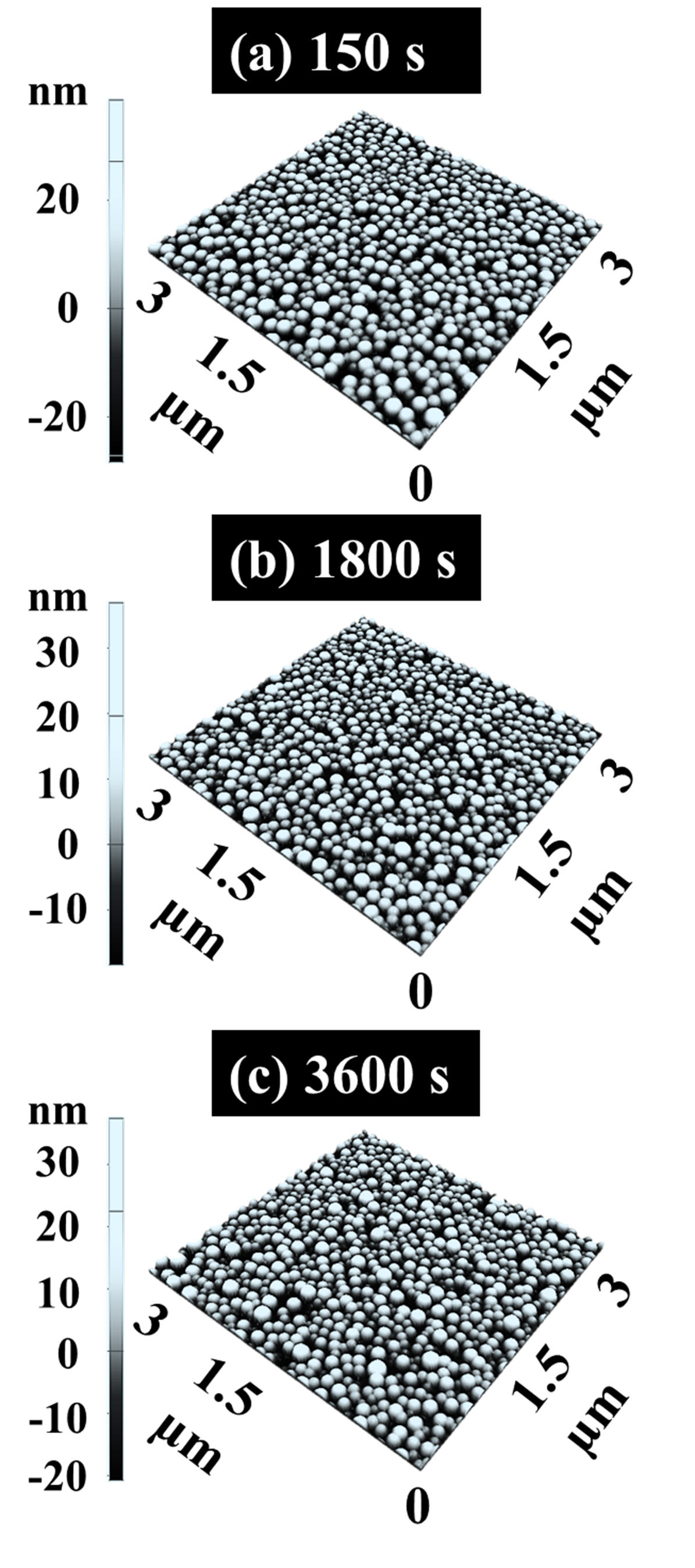

Supplement: Additional file 6: Figure S6. — Fabrication of self-assembled Au NPs by controlling the DT between 150 and 3600 s. (a)–(c) 3-D AFM side views of 3 × 3 μm2. (JPG 350 kb) [file 11671_2015_1200_MOESM6_ESM.jpg]
